# Age-Related Deficits in Electrophysiological and Behavioral Measures of Binaural Temporal Processing

**DOI:** 10.3389/fnins.2020.578566

**Published:** 2020-10-27

**Authors:** Tess K. Koerner, Ramesh Kumar Muralimanohar, Frederick J. Gallun, Curtis J. Billings

**Affiliations:** ^1^VA RR&D National Center for Rehabilitative Auditory Research, VA Portland Health Care System, Portland, OR, United States; ^2^Department of Otolaryngology/Head and Neck Surgery, Oregon Health & Science University, Portland, OR, United States

**Keywords:** aging, electrophysiology, interaural phase difference, binaural processing, IPM-FR, temporal processing, auditory steady-state response, auditory evoked potential

## Abstract

Binaural processing, particularly the processing of interaural phase differences, is important for sound localization and speech understanding in background noise. Age has been shown to impact the neural encoding and perception of these binaural temporal cues even in individuals with clinically normal hearing sensitivity. This work used a new electrophysiological response, called the interaural phase modulation-following response (IPM-FR), to examine the effects of age on the neural encoding of interaural phase difference cues. Relationships between neural recordings and performance on several behavioral measures of binaural processing were used to determine whether the IPM-FR is predictive of interaural phase difference sensitivity and functional speech understanding deficits. Behavioral binaural frequency modulation detection thresholds were measured to assess sensitivity to interaural phase differences while spatial release-from-masking thresholds were used to assess speech understanding abilities in spatialized noise. Thirty adults between the ages of 35 to 74 years with normal low-frequency hearing thresholds were used in this study. Data showed that older participants had weaker neural responses to the interaural phase difference cue and were less able to take advantage of binaural cues for speech understanding compared to younger participants. Results also showed that the IPM-FR was predictive of performance on the binaural frequency modulation detection task, but not on the spatial release-from-masking task after accounting the effects of age. These results confirm previous work that showed that the IPM-FR reflects age-related declines in binaural temporal processing and provide further evidence that this response may represent a useful objective tool for assessing binaural function. However, further research is needed to understand how the IPM-FR is related to speech understanding abilities.

## Introduction

Accurate processing of binaural information is key to sound source localization and the detection of target signals in background noise. One cue used for binaural processing results from differences in the time of arrival of an auditory signal at the two ears. The interaural phase differences (IPDs) between the signal at each ear are detected at the level of the brainstem. The ability to use these binaural IPD cues is dependent on accurate neural firing to the rapid fluctuations in signal amplitude over time, termed temporal fine structure, and the accurate comparison of these temporal cues between the ears. Recent research has shown that aging can impact the ability to process binaural temporal fine structure information independent of hearing loss, resulting in reduced IPD sensitivity (Ross et al., 2007a; Grose and Mamo, 2010, 2012b; Hopkins and Moore, 2011; Gallun et al., 2013, 2014; Papesh et al., 2017; Füllgrabe and Moore, 2018; Vercammen et al., 2018) and deficits understanding speech in background noise (Füllgrabe et al., 2015; Papesh et al., 2017). While the exact cause of this age-related decline in temporal processing is unknown, disruptions in neural synchrony, a slowing of neural activity, a loss of cochlear afferent synapses, deficits in the central integration of binaural information, and/or deficits in the central encoding of binaural information have been known to occur with aging (He et al., 2008; Grose and Mamo, 2010; Ruggles et al., 2012; King et al., 2014; Shaheen et al., 2015; Whiteford et al., 2017; Parthasarathy and Kujawa, 2018; Wu et al., 2019).

Recent reports have focused on the use of non-invasive electrophysiological measures to assess the effects of age on the neural encoding of binaural temporal cues (Ross et al., 2007a; Ross, 2008; Wambacq et al., 2009; Grose and Mamo, 2012a; Ozmeral et al., 2016; Papesh et al., 2017; Eddins and Eddins, 2018; Vercammen et al., 2018; Ungan et al., 2020). For example, Ross et al. (2007a) and Papesh et al. (2017) recorded transient event-related potentials in response to a 180° IPD using magnetoencephalography and electroencephalography, respectively. The IPD cue was embedded in the temporal fine structure of the stimulus by shifting an amplitude modulated (AM) tone from a diotic to a dichotic presentation. This work showed that aging impacted the neural encoding of the IPD cue (Ross et al., 2007a; Papesh et al., 2017) and that age-related changes in neural responses to the IPD cue were associated with individual behavioral limits of IPD discrimination (Ross et al., 2007a), which suggests that this type of measure may represent a robust tool for the neurophysiological assessment of binaural temporal processing abilities.

Recently, there has been growing interest in the use of a new electrophysiological measure called the interaural phase modulation-following response (IPM-FR), which has been developed as a more efficient method to assess the neural encoding of IPDs (Haywood et al., 2015; McAlpine et al., 2016; Undurraga et al., 2016). Similar to neural responses measured by Ross et al. (2007a;b) and Papesh et al. (2017), this response is elicited by a shift in the phase of an AM carrier tone at the two ears. However, rather than a single transient response evoked by a single phase shift, the IPM-FR is a steady-state response to periodic shifts in phase embedded in the temporal fine structure of an ongoing AM tone, which results in a higher number of neural responses over a shorter period of time. This response can be objectively assessed through spectral analysis of the electroencephalographic response in the frequency bin corresponding to the rate at which the phase changes. This provides an additional advantage over the electrophysiological measure used by Papesh et al. (2017), which requires the detection of transient response peaks in the time domain.

To date, only one study has examined whether the IPM-FR is sensitive to the effects of age on binaural temporal processing (Vercammen et al., 2018). Similar to findings from studies that used different electrophysiological measurement techniques (Ross et al., 2007a; Ross, 2008; Ozmeral et al., 2016; Papesh et al., 2017; Ungan et al., 2020), Vercammen et al. (2018) showed that the neural encoding of IPD cues tends to be stronger in younger participants compared to older participants and, along with several other studies (Haywood et al., 2015; Undurraga et al., 2016; Vercammen et al., 2018; Parthasarathy et al., 2020), showed that IPM-FR responses tend to be weaker when behavioral detection of the IPD cue is poor. While these previous studies have established that the IPM-FR is likely reflective of behavioral IPD sensitivity, the stimuli previously used to assess behavioral IPD discrimination thresholds were dichotic AM stimuli analogous to those used to elicit the IPM-FR (Haywood et al., 2015; Undurraga et al., 2016; Vercammen et al., 2018; Parthasarathy et al., 2020). Assessing relationships between the IPM-FR and other behavioral measures of binaural temporal processing will determine whether associations between the IPM-FR and behavior can generalize to other stimuli and tasks that assess IPD sensitivity.

In addition, examining relationships between electrophysiological responses and measures of speech understanding in noise can provide information about neural processes that underlie speech perception deficits. For example, Papesh et al. (2017) showed that variability in neural responses to the IPD cue described above were more predictive of participants’ spatial release-from-masking abilities than age and/or hearing loss. In other words, the electrophysiological measure used by Papesh et al. (2017) was better able to reflect the integrity of binaural temporal processing mechanisms that are important for speech understanding in spatialized noise than participant factors such as age and estimated hearing sensitivity. In addition to recently confirming that the IPM-FR reflects behavioral IPD sensitivity, Parthasarathy et al. (2020) was the first to examine relationships between IPM-FRs and speech perception. Speech understanding was assessed using a competing digits task where a string of digits spoken by target and competing speakers were presented diotically. As expected, the IPM-FR did not represent a link to speech understanding abilities due to the absence of binaural cues in the behavioral task (Parthasarathy et al., 2020). Therefore, to date, it is unknown whether the IPM-FR is also a neural correlate of functional speech understanding abilities in realistic listening environments that require the use of binaural cues. An exploration of additional relationships between neural and behavioral measures will allow for a better understanding of age-related declines in neural processes underlying behavioral measures of binaural function and will provide information about what types of tasks draw upon similar neural resources.

Therefore, while the current study was designed to confirm the effects of age on the IPM-FR, it primarily aimed to evaluate relationships between the IPM-FR and several new behavioral measures of binaural temporal processing to (1) confirm that the IPM-FR is reflective of IPD sensitivity and (2) determine whether variability in the IPM-FR is related to speech understanding in noise abilities. Many common behavioral tests of binaural temporal processing tend to require extensive training periods on the part of the examiner and the listener as well as a high number of stimulus repetitions to obtain reliable estimates of binaural sensitivity (Stecker and Gallun, 2012). These issues have motivated recent research efforts that focus on new implementations of existing laboratory tests which would not require extensive resources, time, or training on the part of the experimenter or participant (Gallun et al., 2013; Füllgrabe and Moore, 2017, 2018; Jakien et al., 2017; Füllgrabe et al., 2018; Jakien and Gallun, 2018; Hoover et al., 2019; Lelo de Larrea-Mancera et al., 2020). Hoover et al. (2019) recently adapted a dichotic frequency modulation (FM) detection task that uses a frequency modulated signal that is inverted in phase at one ear relative to the other to create IPD cues (Grose and Mamo, 2012b; Whiteford et al., 2015). Performance on this task has been shown to be impacted by age, such that older listeners tend to have higher dichotic FM detection thresholds than younger- and middle-aged listeners, reflecting potential age-related declines in temporal fine structure processing (Grose and Mamo, 2012b) or central binaural integration processes (Whiteford et al., 2017). Hoover et al. (2019) showed that, compared to other behavioral tests designed to assess temporal fine structure processing, the FM detection tasks were among the most consistent and efficient measures of binaural processing in a group of young, normal-hearing participants. Several studies have also established the use of a measure of spatial release from masking using speech stimuli for the assessment of binaural function (Gallun et al., 2013; Jakien et al., 2017; Jakien and Gallun, 2018). Spatial release from masking refers to the increased ability to detect a target sound or speech stream of interest when it is spatially separated from one or more maskers (Hawley and Litovsky, 2004; Gallun et al., 2005). Studies have shown that older listeners tend to receive less benefit from the spatial separation of target and background speech streams compared to younger listeners (Gallun et al., 2013; Jakien et al., 2017; Jakien and Gallun, 2018). Spatial release-from-masking thresholds appear to be consistent across different modes of testing, including testing under headphones in a virtual space using traditional laboratory equipment (Jakien et al., 2017) as well as a tablet-based automated rapid-testing version of the task recently developed in the Portable Automated Rapid Testing application (PART; Gallun et al., 2018; Lelo de Larrea-Mancera et al., 2020). Taken together, evidence from these previous studies suggests that measures of binaural FM sensitivity and spatial release from masking may represent efficient and reliable testing tools that are sensitive to age-related changes in binaural function, which motivated their selection for use in the current study.

An additional practical aim of the current study was to explore potential differences in IPM-FRs recorded using different stimulus parameters. Since the stimulus used to elicit the IPM-FR is amplitude modulated, a steady-state response that follows the AM rate occurs in addition to the steady-state neural response to the phase reversal rate. This envelope following response, commonly known as the auditory steady-state response (ASSR), provides a measure of neural phase locking at the place of stimulation equal to the carrier frequency. The ability to concurrently elicit the ASSR along with the IPM-FR is an additional advantage of this electrophysiological measure, as the ASSR may serve as an estimate of hearing sensitivity (Dimitrijevic et al., 2002; Picton et al., 2003), temporal envelope processing, as well as an index of recording and response quality. Previous studies that have examined the IPM-FR have used stimuli that were amplitude modulated at lower (∼40 Hz; Haywood et al., 2015; Undurraga et al., 2016; Parthasarathy et al., 2020) and higher (∼80 Hz; Vercammen et al., 2018) rates. Although still a source of ongoing investigation (Coffey et al., 2019), it is generally thought that ASSRs elicited using a higher AM rate are primarily generated from more subcortical brainstem structures, including the superior olivary complex and inferior colliculus, while ASSRs elicited using a lower AM rate activate overlapping brainstem structures as well as additional neural generators located in the auditory cortex (Giraud et al., 2000; Herdman et al., 2002; Korczak et al., 2012). To date, no studies have directly compared IPM-FRs elicited using different modulation rates in the same individuals. While it is known that changing the AM rate of the stimulus impacts the strength of the ASSR (Levi et al., 1993), it is unknown if, or how, changing the AM rate may impact the IPM-FR. An analysis of this type may provide information regarding the optimization of specific IPM-FR stimulus parameters for a more efficient or reliable assessment of the neural encoding of IPD cues or for a stronger neurophysiological link to behavioral performance.

This work aimed to (1) confirm previous findings that the IPM-FR, measures of binaural FM detection, and measures of spatial release from masking are sensitive to the effects of age on binaural temporal processing; (2) evaluate associations between the IPM-FR and performance on behavioral measures of IPD sensitivity and speech perception; and (3) explore differences in IPM-FRs elicited using different AM rates. It was predicted that, consistent with previous work (e.g., Grose and Mamo, 2012b; Gallun et al., 2013, 2014; Papesh et al., 2017; Füllgrabe and Moore, 2018; Vercammen et al., 2018; Ungan et al., 2020), age would have a significant effect on each neural and behavioral measure of binaural temporal processing, such that older participants would have reduced IPM-FRs, reduced dichotic FM thresholds, and reduced speech understanding abilities compared to younger participants. In addition, it was predicted that older participants would show reduced benefit from the addition of binaural cues in both the FM detection tasks and the spatial release-from-masking tasks compared to younger participants. It was also expected that the strength of the IPM-FR would be predictive of performance on these behavioral measures, suggesting that each measure depends on overlapping neural mechanisms for binaural temporal processing. Finally, while it was unknown whether or not the use of different AM rates would impact the IPM-FR, this manipulation was anticipated to provide important practical guidance on the degree to which AM rate influences IPM-FR strength and its relationship with other measures. Taken together, the results of this work will have important implications for the development and use of measures designed to assess binaural temporal processing abilities.

## Materials and Methods

### Participants

**FIGURE 1:**
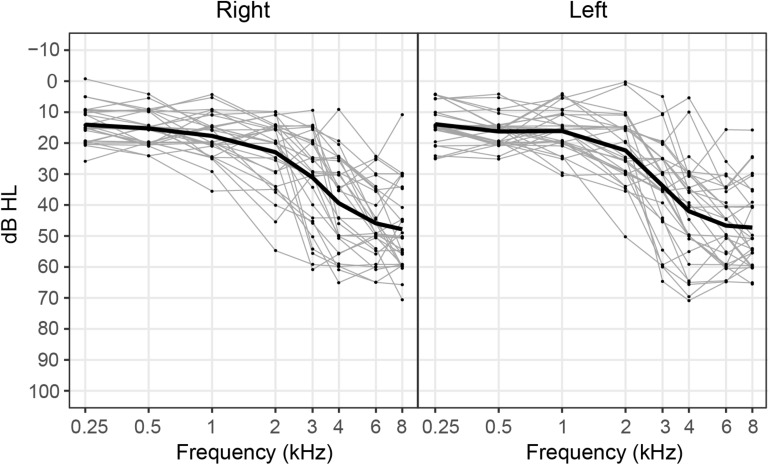
Pure-tone hearing thresholds for each participant (gray lines) and mean hearing thresholds averaged across participants (black line) plotted for the right and left ears. Note that data points depicting individual hearing thresholds are jittered within 1 dB for ease of visualization.

Participants included 30 adults (11 female, 19 male) who ranged in age from 35 to 74 years (mean age: 62.3 years). All participants were right-handed and were native speakers of American English. No participants reported taking medications that impacted sleep or had mood-altering affects. Pure-tone hearing thresholds for each participant as well as mean pure-tone thresholds averaged across participants are depicted for the right and left ears in Figure 1. All participants had hearing thresholds within normal limits (≤25 dB HL) at 500 Hz as measured by a standard pure-tone audiological assessment. This inclusion criterion was specifically chosen because 500 Hz was the carrier frequency used in our neural and behavioral measures of IPD sensitivity. Hearing thresholds at higher frequencies ranged from within normal limits to moderate-to-severe sensorineural hearing loss. No participants had asymmetrical hearing thresholds, as defined as a difference in four-frequency pure-tone averages (0.5, 1, 2, and 4 kHz) greater than 15 dB across ears. All participants provided informed consent prior to their participation in the study and all participants were paid for their participation. This work was approved by the joint Institutional Review Board of the Department of Veterans Affairs Portland Health Care System and Oregon Health & Science University.

### Procedure

#### Neural Measures

**FIGURE 2:**
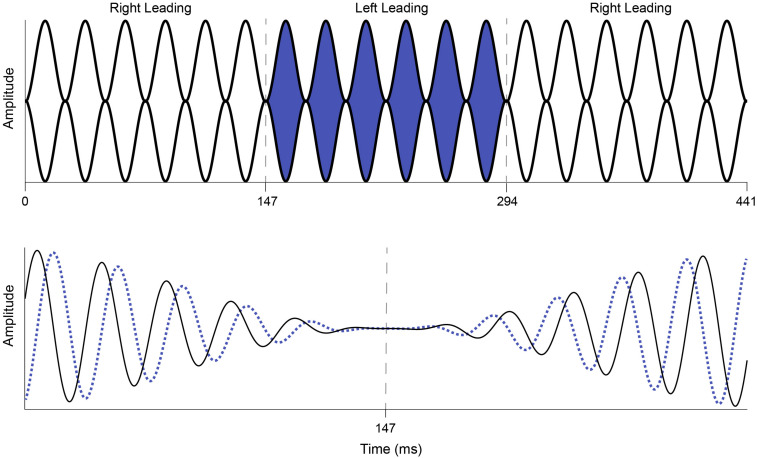
Illustration of the dichotic AM stimulus used to elicit the IPM-FR. The top panel represents a 40.8-Hz AM tone that shifts from right leading (white) to left leading (blue) and back to right leading (white) at an IPM rate of 6.8 Hz. The interaural phase modulations depicted in this figure occur at 147 and 294 ms and are indicated by dashed gray lines. The bottom panel depicts an expanded view of the first phase change in the dichotic AM stimulus shown above. The right ear leads in phase (+90° IPD, solid black line) until the phase switch at 147 ms, at which point the left ear leads in phase (−90° IPD, dotted blue line). The phase change occurs at the zero crossing to avoid audible artifacts.

Interaural phase modulation-following responses were elicited with a 500-Hz tone that was 100% sinusoidally amplitude modulated (AM) at either 40.8 or 81.6 Hz. The modulation envelope of the stimulus remained diotic, but the phase of the carrier frequency was square-wave modulated at a rate of 6.8 Hz such that when the instantaneous phase at one ear was +45°, the phase in the other ear was −45°, creating an IPM depth of ±90°. The 6.8-Hz IPM rate was chosen based on the results of McAlpine et al. (2016), who showed that when compared to slower rates, the 6.8-Hz IPM produced a steady-state neural response and that this neural response was stronger than those elicited by faster IPM rates. Similarly, the single IPM depth of ±90° was chosen because it was previously shown to produce the largest IPM-FR compared to those elicited by higher or lower IPM depths (Haywood et al., 2015; Undurraga et al., 2016). The IPD was introduced at the zero-amplitude minimum in the AM cycle (Figure 2), which helped ensure that neural responses to this stimulus were to shifts in the temporal fine structure, and not due to monaural off-frequency cues (brief broadening of the activation pattern at the level of the cochlea) or modulations in the stimulus envelope. These stimulus parameters are similar to those previously used by Haywood et al. (2015) and Undurraga et al. (2016). The dichotic test stimulus was presented for 5 s and was repeated 75 times per recording block. Seventy-five repetitions of a 5 s diotic control stimulus were also presented in an alternating manner within each recording block with an inter-stimulus interval of 20 ms. This stimulus was identical to the dichotic stimulus described above, except that it had a zero IPD. In other words, phase transitions of equal magnitude occurred at a rate of 6.8 Hz but were the same in both ears. This diotic control stimulus has been used previously to examine the degree to which neural responses at the IPM rate in the dichotic stimulus can be attributed to the introduction of the IPD and not to monaural phase cues (Ross et al., 2007a,b; Haywood et al., 2015; Undurraga et al., 2016). While this diotic control stimulus can serve the same purpose in the current study, it was mainly used to calculate the signal-to-noise ratio (SNR) of the IPM-FR, as described below. Each recording block containing alternating diotic and dichotic stimuli was presented twice, for a total of 150 presentations of each dichotic and diotic stimulus. Separate blocks were recorded at each AM rate, for a total of four recording blocks. The presentation order of each recording block was randomized across participants.

Stimuli were presented bilaterally at 80 dB SPL through Etymotic ER2 insert earphones using Neuroscan software (Compumedics Neuroscan Stim2/Scan 4.5; Charlotte, NC, United States) in an acoustically treated and electrically shielded booth. IPM-FRs were recorded from a 64-channel tin-electrode cap (Electro-Cap International, Inc.; Eaton, OH, United States) with the ground electrode on the forehead and the reference electrode at Cz. Responses were analog low-pass filtered on-line at 200 Hz and were converted using an analog-to-digital sampling rate of 1,000 Hz. Application and preparation of the electrode cap, along with IPM-FR recording, lasted approximately 2 h. During recording, participants were seated in a comfortable chair, asked to relax, minimize eye and muscle movements, and watch a movie of their choice with subtitles.

The electrophysiological data was processed in Neuroscan Edit (Neuroscan, 2007). Eye-blink artifacts were corrected offline using eye movement information collected from the horizontal and vertical planes from electrodes located inferior to and at the outer canthi of both eyes. A spatial, singular value decomposition was used to calculate the amount of covariation between a vertical eye channel and each electrode. This vertical eye blink activity was then removed from each electrode on a point-by-point basis. The remaining ongoing response recorded in each condition was then high-pass filtered at 2 Hz and epoched from 0 to 5000 ms relative to stimulus onset. Epochs exceeding ±100 μV were rejected from analysis. The spectrum of each five second epoch was computed using a 10,000 point Fast Fourier Transform (FFT) which provided a resolution of 0.2 Hz. Mean response magnitudes were calculated by vector averaging these FFT values across all epochs in each condition. IPM-FRs were obtained as the spectral magnitude in the 6.8-Hz bin while ASSRs were obtained as the spectral magnitude in the 40.8- or 81.6-Hz bins for each participant from the electrode at the right mastoid (M2). This electrode site was chosen for analysis based on a previous report that IPM-FR and ASSR magnitudes tended to be largest across participants at the right mastoid (Haywood et al., 2015), which is a pattern consistent with neural responses collected in the current study. The magnitude of the neural activity in the 6.8-Hz bin in the diotic condition at electrode M2 was used as a control condition. An estimate of signal-to-noise ratio of the IPM-FR was calculated for each participant using magnitudes in the 6.8-Hz bin in both the dichotic and diotic conditions, using the formula below:

$$\mathrm{dB}\,\mathrm{SNR}=20\,l\,o\,g_{10}\,\left(\frac{V_{d\,i\,c\,h\,o\,t\,i\,c}}{V_{d\,i\,o\,t\,i\,c}}\right)$$

The measured IPM-FR is thought to consist of energy related to the steady-state response to the IPM rate as well as neural activity that is thought to randomly vary in phase and amplitude over time. Therefore, both the magnitude of the response at 6.8 Hz and this SNR metric were used as measures of the IPM-FR in an attempt to better characterize response strength.

#### Behavioral Measures

The behavioral binaural FM detection tasks and spatial release-from-masking tasks were completed using test batteries included in PART (Lelo de Larrea-Mancera et al., 2020) on an iPad with Sennheiser 280 Pro circumaural headphones (Wedemark, Germany). All stimuli were presented at a level of 80 dB SPL and all testing was completed in a sound-treated and electrically shielded booth. Each behavioral task was repeated twice and the presentation order was randomized across participants.

**FIGURE 3:**
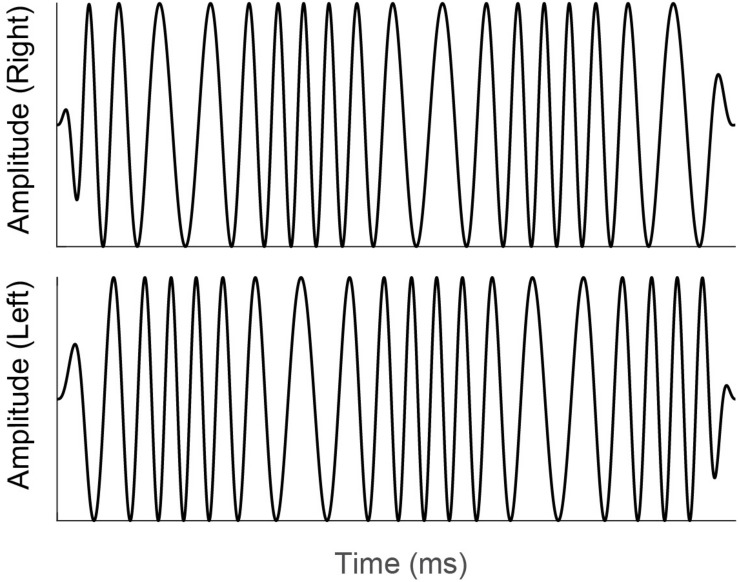
Illustration of a dichotic FM stimulus similar to that used in the current study. Note that monaural FM that is out of phase at the right (top panel) and left (bottom panel) ears creates dynamic interaural time difference cues. Participants were instructed to select the stimulus interval with the interaural time difference cues that was perceived as a signal moving in the head between the two ears.

Diotic and dichotic FM detection thresholds were measured using a four-interval, two-cue, two-alternative forced choice procedure (Hoover et al., 2019; Lelo de Larrea-Mancera et al., 2020). Each standard stimulus was an unmodulated pure tone and the target stimulus was a pure tone carrier with a 6.8-Hz sinusoidal modulator that was presented in either the 2nd or 3rd stimulus interval on a touchscreen iPad display. This modulation rate was chosen to match the modulation rate of the stimulus used to elicit the IPM-FR, described above. All standard and target stimulus presentations were 400 ms long and had carrier frequencies that were randomly selected from a uniform distribution that ranged from 460 to 540 Hz. Randomizing the carrier frequency in this way lessens the possibility that participants use a place cue to detect the frequency modulated target stimulus from the unmodulated pure-tone standard stimuli. All stimuli had inter-stimulus intervals of 250 ms. Modulation depth was adaptively varied in logarithmic steps using the algorithm described in Lelo de Larrea-Mancera et al. (2020). This staircase procedure estimates the lowest rate, in Hz, at which a given individual can just detect the frequency modulation. In the diotic testing condition, the target FM signal was the same across both ears with a starting phase of 0 radians. Participants were instructed to detect the interval with the frequency modulated, “warbling” stimulus. In the dichotic testing condition, the target stimulus contained monaural FM that was out of phase at the two ears, with modulator starting phases of 0 and π radians. The resulting interaural phase modulation created dynamic interaural time difference (ITD) cues which created the percept of the signal moving in the head between the two ears at a rate of 6.8 Hz. The dichotic FM threshold, in Hz, corresponds to the smallest ITD that can be detected in this stimulus. An example of a dichotic FM stimulus is provided in Figure 3. Note that to improve visualization of the dichotic FM, the stimulus depicted in Figure 3 has a lower carrier frequency and higher modulation rate compared to the dichotic FM stimulus used in the current study. Participants were not given any practice trials for either the diotic FM or dichotic FM conditions. Participants received feedback on each trial that indicated whether their selection was correct or incorrect. Detection thresholds were log transformed. The better of the two diotic FM detection thresholds and the better of the two dichotic FM detection thresholds were chosen for analysis. In addition, a difference score was calculated to estimate benefit from the addition of binaural cues for FM detection. For this calculation, the difference in individual dichotic FM and diotic FM detection thresholds was taken relative to individual performance in the diotic FM condition (Grose and Mamo, 2012b).

Spatial release from masking was measured using sentences from three male speakers from the Coordinate Response Measure (Bolia et al., 2000). The target and masker sentences were presented in colocated and spatially separated listening conditions, which were used to calculate spatial release from masking (Gallun et al., 2013; Jakien et al., 2017). Sentences were of the form: “Ready (CALL SIGN) go to (COLOR) (NUMBER) now.” Participants were instructed to choose the appropriate color and number combination associated with the call sign “Charlie,” which was always spoken by the talker located at 0°. Participants made choices on a color-number grid that was presented on the touchscreen iPad display. Distractor speakers each used one of seven different callsigns, such as “Eagle” or “Baron,” and different color number combinations from those spoken by the target speaker. The distractors were located at +45° and −45° in the separated listening condition and at 0° in the more difficult colocated listening condition. The locations of the target talker and each distractor talker were simulated by convolving the anechoic sentences with the head-related impulse responses measured at those locations in the horizontal plane (see Gallun et al., 2013). To familiarize participants with the response format prior to testing, a short practice session was provided in which the target “Charlie” sentences were spoken without any competing distractor speakers. Progressive tracking was used in each testing repetition as described in Gallun et al. (2013), which involves reducing the target-to-masker ratios from 10 to −8 dB in 2 dB steps. Participants were given two trials at each target-to-masker ratio. Feedback was provided on each trial to indicate whether the response was correct or incorrect. Target-to-masker thresholds (in dB), which approximate the point at which performance is 50%, were calculated by subtracting the number of correct responses from the starting target-to-masker ratio of 10 dB (see Gallun et al., 2013 for further details). Thresholds for the separated and colocated conditions were averaged across the two separate testing repetitions for each participant. Spatial release from masking, in dB, was calculated as the difference in threshold from the colocated to the spatially separated listening condition.

### Analysis

Separate linear regression models were created in R (R Core Team) using the *nlme* package (Pinheiro et al., 2016) to assess the effects of age and hearing sensitivity on each test measure, with α = 0.05. Either participant age or hearing sensitivity were added as a fixed effect while separate models were used to evaluate the various outcome measures: IPM-FR magnitude or SNR in each AM rate condition, diotic FM or dichotic FM detection threshold and binaural FM difference score, and target-to-masker ratio threshold and spatial release-from-masking threshold. The effects of age and hearing sensitivity on the ASSR magnitude in each recording condition were also assessed to determine the potential effects of each factor on this neural response. Hearing sensitivity was estimated by averaging hearing thresholds across frequencies and ears to account for potential effects of variations in high frequency hearing sensitivity across participants.

Similar linear regression models were created to assess relationships between the neural measures and performance on the binaural FM and spatial release-from-masking measures. In these models, either IPM-FR magnitude or SNR from each AM rate condition was added as a fixed effect to predict each behavioral outcome measure. Paired *t*-tests, with α = 0.05, were used to assess potential effects of AM rate on the IPM-FR magnitude and SNR. Similarly, paired *t*-tests were used to assess the effects of stimulus condition (diotic vs. dichotic) on ASSR magnitude. Since the AM remained diotic in each stimulus condition, a comparison of ASSR magnitude to the dichotic test stimulus which contained IPDs and the diotic test stimulus which did not contain IPDs would assess the degree to which the recording and response quality remained comparable across alternating stimulus repetitions.

## Results

### Effects of Age and Hearing Sensitivity on Neural and Behavioral Measures

Participant age was not significantly associated with hearing sensitivity as measured by an average of pure-tone thresholds across frequency and ears [*F*(1,28) = 2.79, *p* = 0.11, *R*^2^ = 0.09] or as measured by 500-Hz thresholds averaged across ears [*F*(1,28) = 1.13, *p* = 0.30, *R*^2^ = 0.04].

#### Neural Measures

**FIGURE 4:**
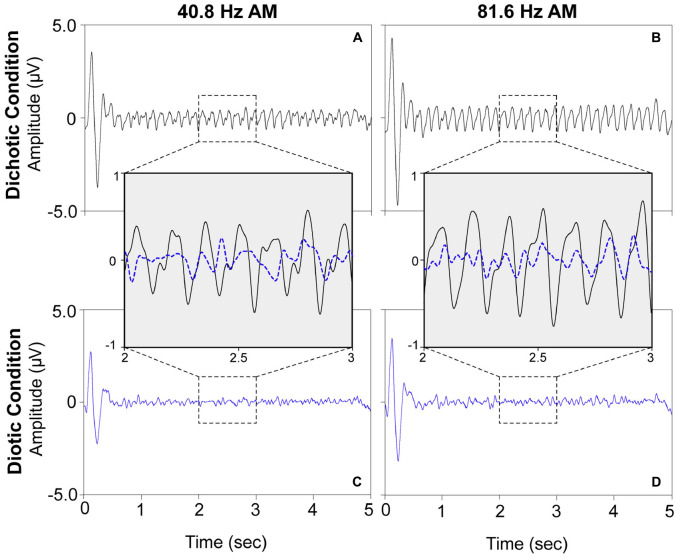
Grand mean time-domain response waveforms for each stimulus condition averaged across all 30 participants at electrode M2. The dichotic test stimuli elicited steady-state neural responses at the IPM rate of 6.8 Hz (panels **A,B**) which are not observed in response to the diotic control stimuli (panels **C,D**). A direct comparison of a 1 s segment of the neural responses to the dichotic (black) and diotic (dotted blue) stimuli are provided in inset panels for the 40.8-Hz AM stimuli (left) and the 81.6-Hz AM stimuli (right). For visualization of the IPM-FR in the time domain, the ongoing EEG signal was bandpass filtered from 2–20 Hz.

**FIGURE 5:**
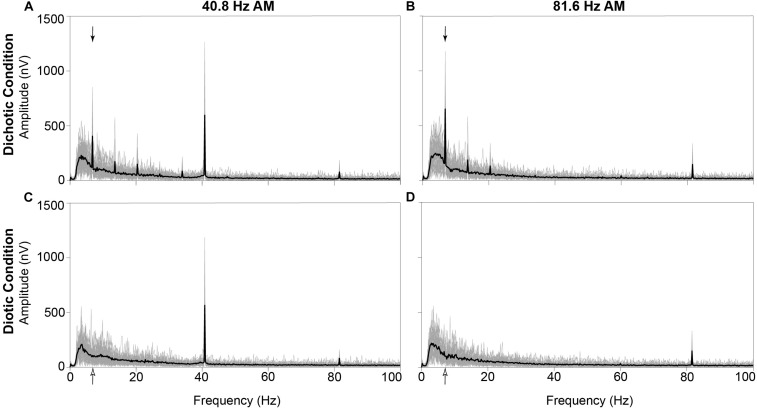
Grand mean neural responses in the frequency domain averaged across all 30 participants (black line) and individual neural response spectra from each participant (gray lines) at electrode M2. The IPM-FR is clearly observed as a peak at 6.8 Hz in response to the dichotic test stimuli (denoted by filled arrows in panels **A,B**). As expected, this response peak is not observed at 6.8 Hz in response to the diotic control stimuli (6.8-Hz bin denoted by unfilled arrows in panels **C,D**). In addition, ASSRs are observed at 40.8 Hz (panels **A,C**) and 81.6 Hz (panels **B,D**) in response to both the dichotic and diotic test stimuli.

For visualization purposes, grand mean time-domain response waveforms are shown in Figure 4 for each stimulus condition. The IPM-FR is observed as the steady-state neural response that follows the 6.8-Hz IPM rate in the dichotic test conditions. As expected, this steady-state response is absent in the diotic control conditions which contained no IPDs. While the IPM-FR can be observed in the time-domain, it is more easily examined and measured in the frequency domain. Grand mean response spectra averaged across each participant are provided in Figure 5. Figure 5 also includes individual response spectra to illustrate the range of IPM-FR and ASSR magnitudes recorded across participants^1^. The IPM-FR is clearly observed as a response peak at 6.8 Hz in each dichotic test condition (indicated by the arrows in Figures 5A,B). Harmonics of the 6.8-Hz IPM rate can also be observed in each dichotic test condition. In addition, the ASSR can be observed as a response peak corresponding to the AM rate of each stimulus. The IPM-FR response peak at 6.8 Hz and subsequent harmonics are absent in the diotic control conditions for both stimuli, but ASSRs are still observed at the 40.8- and 81.6-Hz AM rates (Figures 5C,D, respectively). Figures 4, 5 are provided for the visualization of example IPM-FRs and ASSRs and to illustrate general response trends for each stimulus condition. Given that the main purpose of this study was to examine the effects of age on binaural temporal processing using the IPM-FR and to examine relationships between neural responses and behavioral performance, individual neural responses were used for all statistical analyses.

**FIGURE 6:**
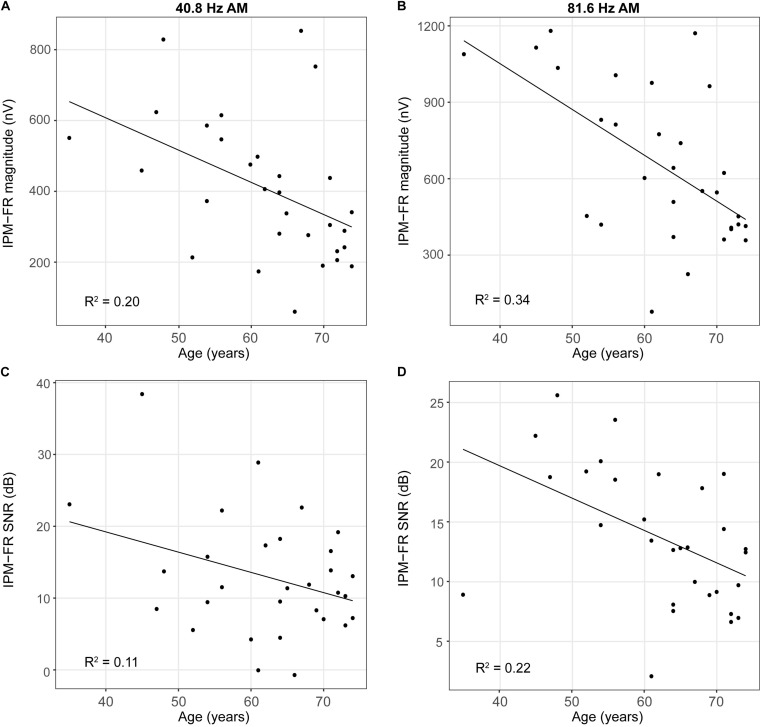
Scatterplots depicting relationships between participant age and IPM-FR magnitude (panels **A,B**) and between participant age and IPM-FR SNR (panels **C,D**). Please note differences in the range of IPM-FR magnitudes displayed in Panels **(A)** and **(B)** and differences in the range of IPM-FR SNR values displayed in Panels **(C)** and **(D)**.

Results from the linear regression models that were used to assess the effects of age or hearing sensitivity on each neural measure are provided in Table 1. Analysis indicated significant effects of age on IPM-FR magnitude in the 40.8-Hz AM rate condition [*F*_(__1_,_28__)_ = 7.17, *p* = 0.01, *R*^2^ = 0.20] and significant effects of age on both IPM-FR magnitude [*F*_(__1_,_28__)_ = 14.67, *p* < 0.001, *R*^2^ = 0.34] and SNR [*F*_(__1_,_28__)_ = 7.92, *p* = 0.009, *R*^2^ = 0.22] in the 81.6 Hz-AM rate condition. For these measures, older individuals tended to have weaker IPM-FRs compared to younger individuals (Figure 6). The lack of an age-related effect on response magnitude in the 6.8-Hz bins in the diotic control conditions suggests that age did not impact estimates of background noise. Variability in hearing sensitivity did not have a significant effect on any neural measure.

**TABLE 1:** Test statistics from linear regression models that examined the effects of participant age and average hearing sensitivity across frequency on each neural measure.

**Neural measure**	**AM Rate (Hz)**	**Age**			**Hearing sensitivity**		
		***F***	***p***	***R*^2^**	***F***	***p***	***R*^2^**
IPM-FR Magnitude (nV): Dichotic Condition	40.8	7.17	0.01	0.20	0.05	0.82	0.00
	81.6	14.67	<0.001	0.34	1.05	0.31	0.04
Control Magnitude (nV): Diotic Condition	40.8	0.15	0.70	0.00	0.01	0.92	0.00
	81.6	0.02	0.89	0.00	1.37	0.25	0.05
IPM-FR SNR (dB)	40.8	3.48	0.07	0.11	0.27	0.61	0.01
	81.6	7.92	0.009	0.22	0.43	0.52	0.01

Additional analyses were completed to assess the potential effect of age on the ASSR in each test condition. For the 40.8-Hz AM rate condition, age had a significant effect on ASSR magnitude in both the dichotic [*F*(_1_,_28__)_ = 10.85, *p* = 0.003, *R*^2^ = 0.28] and diotic [*F*(_1_,_28__)_ = 12.26, *p* = 0.002, *R*^2^ = 0.30] test conditions, such that older individuals had smaller ASSR magnitudes compared to younger participants. These age-related changes in ASSR magnitudes were not present in either the dichotic or diotic test conditions when the higher 81.6-Hz AM stimulus was used.

#### Behavioral Measures

**FIGURE 7:**
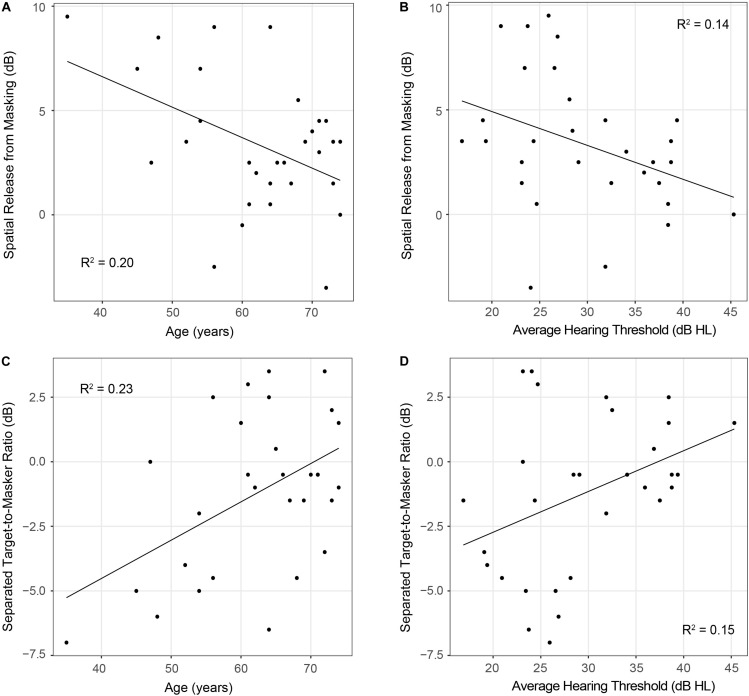
Scatterplots depicting relationships between participant age (panels **A,C**) or average hearing thresholds (panels **B,D**) and performance on spatial release-from-masking measures. Note that lower spatial release-from-masking thresholds represent poorer performance on this measure and indicate that participants had smaller differences in performance between the colocated and separated speech-on-speech masking conditions. On the other hand, in the spatially separated speech-on-speech masking condition, lower thresholds represent better performance and indicate that participants were able to identify the target sentence at more difficult target-to-masker ratios than participants with higher thresholds.

Results from the linear regression models that were designed to examine the effects of age and hearing sensitivity on each behavioral measure are provided in Table 2. This analysis revealed a significant effect of age on diotic FM detection [*F*_(__1_,_28__)_ = 4.40, *p* = 0.04, *R*^2^ = 0.14] such that older individuals had higher FM detection thresholds, and therefore poorer performance on this task compared to younger individuals. However, results did not indicate that age had a significant effect on performance in the dichotic FM condition or on the FM difference score. Both age and hearing sensitivity had significant effects on target-to-masker ratio thresholds in the separated listening condition of the spatial release-from-masking task [Age: *F*_(__1_,_28__)_ = 8.44, *p* = 0.007, *R*^2^ = 0.23; Hearing Sensitivity: *F*_(__1_,_28__)_ = 4.79, *p* = 0.04, *R*^2^ = 0.15]. Neither age nor hearing sensitivity had a significant impact on performance in the more difficult colocated condition of this task where the distractor speakers were located at the same azimuth as the target speaker. When performance on these two listening conditions were compared, analyses revealed a significant impact of both age and hearing sensitivity on spatial release-from-masking thresholds [Age: *F*_(__1_,_28__)_ = 7.04, *p* = 0.01, *R*^2^ = 0.20; Hearing Sensitivity: *F*_(__1_,_28__)_ = 4.45, *p* = 0.04, *R*^2^ = 0.14]. For these measures, participants who were older or who had poorer average across-frequency hearing thresholds required more favorable target-to-masker ratios to obtain 50% performance (Figure 7).

**TABLE 2:** Test statistics from linear regression models that examined the effects of participant age and average hearing sensitivity across frequency on each behavioral measure.

**Behavioral measure**	**Age**			**Hearing sensitivity**		
	***F***	***p***	**R^2^**	***F***	***p***	**R^2^**
**Binaural FM Detection Thresholds**						
Dichotic Condition (log Hz)	2.03	0.16	0.07	0.07	0.79	0.00
Diotic Condition (log Hz)	4.40	0.04	0.14	0.03	0.87	0.00
Difference Score	1.83	0.19	0.06	0.00	0.99	0.00
**Spatial Release-from-Masking Thresholds (dB)**						
Separated Condition	8.44	0.007	0.23	4.79	0.04	0.15
Colocated Condition	0.01	0.93	0.00	0.01	0.91	0.00
Spatial Release from Masking	7.04	0.01	0.20	4.45	0.04	0.14

### Relationships Between Neural Responses and Behavioral Performance

Linear regression models were used to test relationships between the IPM-FR and performance on behavioral measures of binaural processing. Results from these regression models are provided in Tables 3, 4.

**TABLE 3:** Test statistics from linear regression models that examined relationships between each IPM-FR measure and each binaural FM measure.

		**Dichotic FM (log Hz)**			**Diotic FM (log Hz)**			**FM Difference Score**		
		***F***	***p***	**R^2^**	***F***	***p***	**R^2^**	***F***	***p***	**R^2^**
IPM-FR Magnitude (nV)	40.8	1.91	0.18	0.06	4.12	0.05	0.13	2.64	0.11	0.09
	81.6	3.72	0.06	0.12	4.05	0.05	0.13	3.11	0.09	0.10
SNR (dB)	40.8	1.79	0.20	0.06	1.86	0.18	0.06	2.06	0.16	0.07
	81.6	16.55	<0.001	0.37	4.34*	0.05	0.13	18.76	<0.001	0.40

*Denotes relationships that were no longer statistically significant after accounting for the effects of age or average hearing sensitivity on behavioral performance.

**TABLE 4:** Test statistics from linear regression models that examined relationships between each IPM-FR measure and target-to-masker ratio thresholds in the separated and colocated speech-on-speech masking conditions as well as spatial release-from-masking thresholds.

		**Separated Condition (dB)**			**Colocated Condition (dB)**			**Spatial Release from Masking (dB)**		
		***F***	***p***	**R^2^**	***F***	***p***	**R^2^**	***F***	***p***	**R^2^**
IPM-FR Magnitude (nV)	40.8	2.90	0.10	0.09	0.36	0.55	0.01	1.76	0.19	0.06
	81.6	7.43*	0.01	0.21	0.33	0.57	0.01	4.98*	0.03	0.15
SNR (dB)	40.8	1.74	0.20	0.06	0.80	0.38	0.03	0.73	0.40	0.03
	81.6	2.73	0.11	0.09	1.36	0.25	0.05	1.08	0.31	0.04

*Denotes relationships that were no longer statistically significant after accounting for the effects of age or average hearing sensitivity on behavioral performance.

#### Binaural FM Detection

**FIGURE 8:**
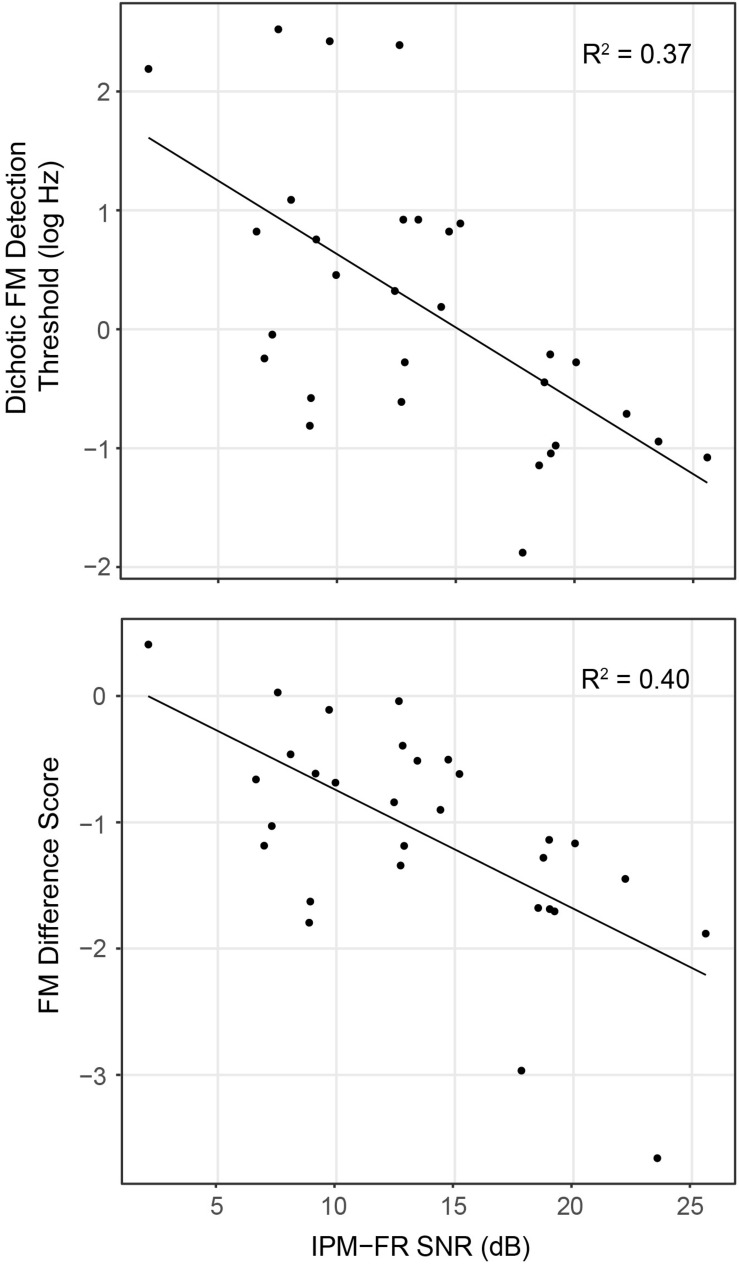
Scatterplots depicting relationships between the IPM-FR SNR recorded in response to the 81.6-Hz AM stimulus for each participant and individual dichotic FM detection thresholds (top panel) and FM difference score estimates (bottom panel). Note that lower dichotic FM thresholds represent better performance on this task, while lower (more negative) FM difference scores indicate a greater difference between diotic and dichotic FM thresholds, representing a larger benefit from the addition of binaural information in this task.

Analysis showed that the IPM-FR SNR in the 81.6-Hz AM condition was significantly predictive of dichotic FM detection thresholds [*F*_(__1_,_28__)_ = 16.55, *p* < 0.001, *R*^2^ = 0.37] and the FM difference score [*F*_(__1_,_28__)_ = 18.76, *p* < 0.001, *R*^2^ = 0.40]. Relationships between the IPM-FR SNR and these binaural FM measures are depicted in Figure 8, which shows that weaker IPM-FRs are associated with poorer behavioral IPD sensitivity as well as less benefit from the addition of binaural information to the FM detection task. Adding either participant age or estimated hearing sensitivity to these models did not predict any additional variance in performance. The IPM-FR SNR in the 81.6-Hz AM rate condition was also predictive of diotic FM detection thresholds [*F*_(__1_,_28__)_ = 4.34, *p* = 0.046, *R*^2^ = 0.13]. However, further analyses revealed that this relationship was primarily mediated by the effect of age. When the effects of age were accounted for in the model, the relationship between the IPM-FR SNR and diotic FM thresholds was no longer significant [*F*_(__1_,_27__)_ = 1.58, *p* = 0.22]. IPM-FR magnitudes in either AM rate condition and IPM-FR SNRs in the 40.8-Hz AM condition were not related to performance on any binaural FM measure.

#### Spatial Release From Masking

**FIGURE 9:**
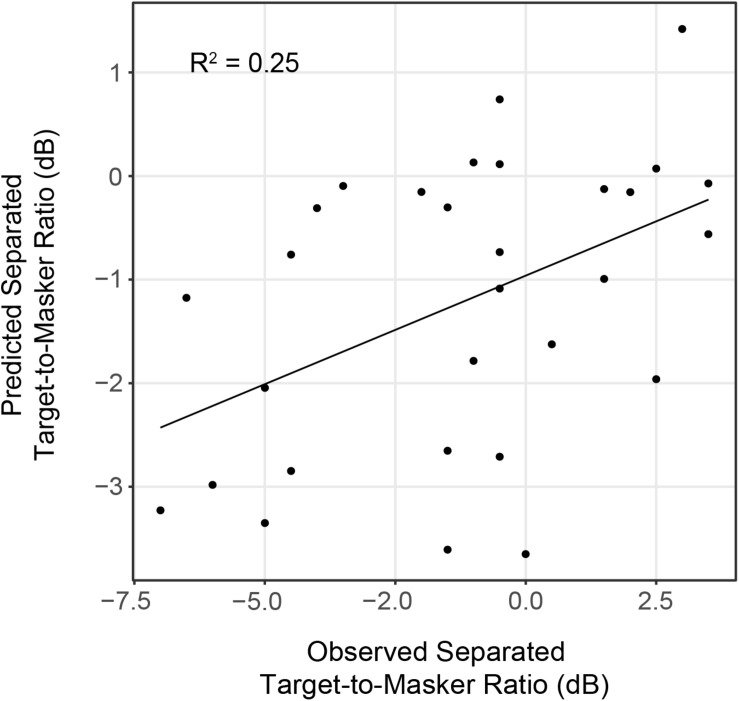
Observed target-to-masker ratio thresholds in the spatially separated listening condition plotted as a function of predicted thresholds from a linear regression model that included average hearing threshold and IPM-FR magnitude to the 81.6-Hz AM stimulus condition as fixed effects.

Linear regression analyses revealed that IPM-FR magnitude in the 81.6-Hz AM condition was significantly predictive of target-to-masker ratio thresholds in the separated listening condition [*F*_(__1_,_28__)_ = 7.43, *p* = 0.01; *R*^2^ = 0.21] and spatial release-from-masking thresholds [*F*_(__1_,_28__)_ = 4.98, *p* = 0.03, *R*^2^ = 0.15]. The combination of average hearing threshold and IPM-FR magnitude as fixed effects in the model accounted for even more variability in target-to-masker ratio thresholds in the separated listening condition [*F*_(__2_,_27__)_ = 5.79, *p* = 0.008, adjusted *R*^2^ = 0.25] than IPM-FR magnitude alone (Figure 9). However, further analysis revealed that IPM-FR magnitude was not a significant predictor of these target-to-masker ratio thresholds when participant age was included in the model. Similarly, IPM-FR magnitude did not explain any additional variance in spatial release-from-masking thresholds than the addition of participant age and average hearing thresholds alone [*F*_(__2_,_27__)_ = 4.79, *p* = 0.02, adjusted *R*^2^ = 0.21]. In other words, the significant relationships between IPM-FR magnitude and performance on these spatial release-from-masking tasks may be primarily mediated by the effects of age. No other IPM-FR measures were significantly associated with performance on the spatial release-from-masking tasks.

### Comparison of IPM-FRs by AM Rate

**FIGURE 10:**
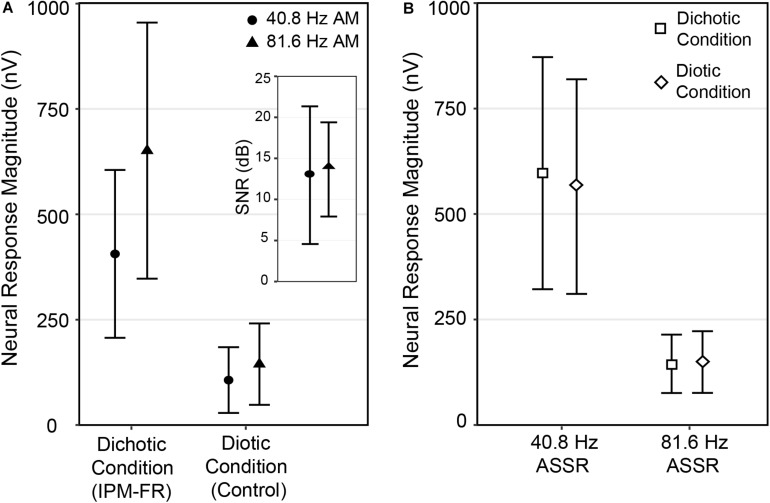
Mean neural response magnitudes in the dichotic (IPM-FR) and diotic (control) conditions for the 40.8-Hz AM (filled circles) and the 81.6 Hz AM (filled triangles) stimuli (panel **A**) averaged across all 30 participants at electrode M2. The inset figure shows mean IPM-FR SNRs for the 40.8-Hz AM stimulus (filled circles) and the 81.6-Hz AM stimulus (filled triangles) calculated using individual neural responses to the dichotic and diotic stimuli. Mean ASSR magnitudes recorded in the dichotic (open square) and diotic (open diamond) stimulus are also provided (panel **B**). Error bars represent 1 standard deviation from the mean.

Mean neural response magnitudes and standard deviations for each electrophysiological measure are plotted in Figure 10. Paired *t*-tests were completed to determine whether IPM-FR magnitude and SNR were significantly different in response to different AM rates. Analysis showed that the magnitude of the IPM-FR was significantly larger in the 81.6-Hz AM rate condition compared to the 40.8-Hz AM rate condition [*t*(1,29) = 7.60, *p* < 0.001]. However, there was no significant difference in SNRs between the two stimulus conditions [*t*(1,29) = 0.46, *p* = 0.65]. In addition, there was no significant difference between neural responses in the diotic control condition at 6.8 Hz across the two AM rates [*t*(1,29) = 1.65, *p* = 0.11].

### Comparison of ASSRs by Test Condition

Paired *t*-tests were also completed to determine whether ASSR magnitudes were significantly different across dichotic and diotic recording conditions. This analysis revealed that there was a significant effect of test condition (diotic vs. dichotic) on the 40.8-Hz ASSR such that ASSR magnitude was significantly higher in the dichotic condition compared to the diotic control condition [*t*(1,29) = 2.63, *p* = 0.01]. In contrast, the magnitude of the 81.6-Hz ASSR was not significantly impacted by test condition [*t*(1,29) = −0.65, *p* = 0.52].

## Discussion

The current study aimed to confirm the effects of age on the electrophysiological IPM-FR and behavioral measures of binaural temporal processing (binaural FM detection and spatial release from masking). In addition, this work was designed to determine whether age-related variability in the neural encoding of IPD cues, as measured by the IPM-FR, is related to performance on each behavioral task. Finally, the current study also aimed to explore potential differences in IPM-FRs measured using stimuli with different AM rates.

### Effects of Age and Hearing Sensitivity on Binaural Processing

This study showed that age had a significant effect on IPM-FR magnitude and SNR. These findings are consistent with previous work (Vercammen et al., 2018) and provide additional evidence that age can impact the neural encoding of IPD cues (Ross et al., 2007a; Grose and Mamo, 2012a; Ozmeral et al., 2016; Papesh et al., 2017; Eddins and Eddins, 2018; Ungan et al., 2020). The IPM-FR is measured in response to IPD cues from temporal fine structure differences in an ongoing stimulus and is likely impacted by deficits in the extraction and integration of IPD information at the level of the brainstem. However, the IPM-FR is thought to be generated from neurons in the auditory cortex (Dajani and Picton, 2006; Undurraga et al., 2016). Therefore, it is likely that this response also reflects the cortical encoding of IPD cues. Although this work is unable to disentangle the potential effects of age on these subcortical and/or cortical processes, the current results do suggest that the IPM-FR represents a robust tool for the assessment of age-related declines in the neural processing of IPD cues.

Interestingly, while age did have a significant impact on diotic FM detection thresholds, it did not have a significant effect on dichotic FM detection thresholds or FM difference scores, which are thought to reflect IPD processing abilities. This is inconsistent with findings from Grose and Mamo (2012b), who showed that dichotic FM detection thresholds were better able differentiate between participant age groups than diotic FM detection thresholds. In addition, this previous work showed that younger and middle-aged participants were able to obtain more benefit from the addition of binaural information provided in the dichotic FM condition compared to older participants (Grose and Mamo, 2012b). Discrepancies between findings from the current study and those of Grose and Mamo (2012b) may be related to differences in stimulus modulation rates and durations. The current study used a 400-ms stimulus with a 6.8-Hz modulation frequency to match the IPM rate used in the electrophysiological measure, while Grose and Mamo (2012b) used a 1025-ms stimulus with a lower 2-Hz modulation frequency. The higher rate used in the current study resulted in more cycles of modulation per stimulus presentation over a shorter period of time compared to the stimulus used by Grose and Mamo (2012b). Increasing the number of modulation cycles has been shown to improve FM detection thresholds in both monaural (Hartmann and Klein, 1980; Wallaert et al., 2018; Palandrani et al., 2020) and dichotic listening conditions (Palandrani et al., 2020). Grose and Mamo (2012b) reported mean dichotic FM detection thresholds of approximately 2 Hz for their group of older (65–77 years) listeners and approximately 0.8 Hz for their group of middle aged (43–57 years) listeners, for an estimated average threshold across groups of approximately 1.4 Hz. This estimated threshold is slightly better than the mean dichotic FM detection threshold of 1.6 Hz in the current study, which tested a comparable group of listeners who ranged in age from 35–74 years. While it appears as if participants from Grose and Mamo (2012b) and those tested in the current study performed similarly on this task, differences in modulation rates used across studies makes these threshold comparisons difficult. Instead, as shown in Witton et al. (2000), thresholds can be converted to ITDs for a given modulation depth (or FM detection threshold), modulation rate, and center frequency to directly compare performance across studies. When compared in this way, the 1.4-Hz detection threshold from Grose and Mamo (2012b) corresponds to a maximum difference ITD of approximately 891.3 μs, while the 1.6-Hz detection threshold found in the current study corresponds to a maximum difference ITD of 299.6 μs, representing better performance on this task. It is possible that increasing the modulation rate reduces the difficulty of the dichotic FM detection task and consequently impacts this measure’s sensitivity to the effects of age on binaural processing, giving rise to the discrepancy between the current results and those of Grose and Mamo (2012b). Future research should focus on further exploring the effects of age and modulation rate on IPD sensitivity using this dichotic FM detection task.

Participants in the current study were required to have hearing thresholds within normal limits (i.e., ≤25 dB HL) at 500 Hz. Variations in hearing sensitivity at higher frequencies were not expected to have any impact on the binaural FM measures or the IPM-FR, as a 500 Hz carrier tone was used as a stimulus for each of these measures. However, variability in higher frequency hearing thresholds may indicate differences in the overall health of the auditory system that could impact auditory processing, including the encoding and detection of low-frequency IPD cues. In order to account for this possibility, hearing sensitivity was characterized as the average of hearing thresholds from 250 to 8000 Hz across ears. This metric accounts for variability in low-frequency hearing thresholds as well as variability in high-frequency hearing thresholds across participants. The current results did not show any effect of hearing sensitivity on the IPM-FR or the binaural FM detection measures. Previous studies have not shown significant effects of high-frequency hearing sensitivity on low-frequency IPD discrimination using a variety of behavioral measures (Strelcyk and Dau, 2009; Grose and Mamo, 2012b; Moore et al., 2012; Eddins and Eddins, 2018), which is consistent with the current results. However, Vercammen et al. (2018) did show that hearing sensitivity impacted the neural encoding of IPDs as measured by IPM-FRs elicited by a 492-Hz carrier tone, even after presentation levels were adjusted for audibility. Unlike the current study that treated age as a continuous variable, Vercammen et al. (2018) separated participants into younger-, middle-, and older-aged normal hearing and hearing impaired participant groups, which differed in average hearing thresholds at 500 Hz. This may have resulted in different levels of stimulus audibility and therefore different presentation levels across these participant groups, which may have contributed to the main effect of hearing sensitivity reported by Vercammen et al. (2018) that was not observed in the current study.

Even though variability in high-frequency hearing thresholds was not expected to impact low-frequency IPD sensitivity, results showed that hearing sensitivity did have a significant effect on spatial release-from-masking thresholds and performance in the spatially separated speech-on-speech listening condition. It is likely that poorer high-frequency hearing sensitivity impacted the audibility of certain speech cues necessary for this behavioral task. Results showed that age also had a significant effect on these measures and had a stronger relationship with speech understanding than average hearing sensitivity. This is consistent with previous findings that have shown that both age and hearing sensitivity can independently impact performance on these spatial release-from-masking measures (Gallun et al., 2014; Papesh et al., 2017; Jakien and Gallun, 2018).

Since the ASSR reflects phase locking to the temporal envelope of the amplitude modulated stimulus, this electrophysiological measure can represent an index of temporal processing abilities. The current study showed that age had a significant impact on the 40.8 Hz ASSR recorded in each test condition, but did not significantly impact the 81.6 Hz ASSR. This finding suggests that in addition to impacting binaural temporal fine structure processing, age may also affect temporal envelope processing abilities. This finding is consistent with results from Ungan et al. (2020), who used a binaural beat stimulus and an amplitude modulated stimulus to examine the effects of age on the neural encoding of binaural temporal fine structure information and on the neural encoding of temporal envelope information, respectively. As discussed previously, it is thought that higher amplitude modulation rates elicit ASSRs from brainstem structures, while lower amplitude modulation rates elicit ASSRs that may be generated by contributions from overlapping brainstem as well as cortical structures (Giraud et al., 2000; Herdman et al., 2002; Korczak et al., 2012). Therefore, the current results may reflect age-related declines in the cortical processing of temporal envelope information that is not reflected at the level of the brainstem. However, this pattern of results is inconsistent with previous literature that has not shown any significant effects of age on 40 Hz ASSRs (Johnson et al., 1988; Boettcher et al., 2001; Picton et al., 2003; Ross, 2008). In fact, several studies have actually shown that age tends to have a greater impact on ASSRs elicited by higher AM rates compared to lower AM rates (Leigh-Paffenroth and Fowler, 2006; Grose et al., 2009; Goossens et al., 2016). It is unknown why the current ASSR results followed an opposite pattern.

### Relationships Between Neural Responses and Behavioral Performance

Relationships between binaural FM thresholds and the IPM-FR were examined in order to better understand whether this electrophysiological measure is reflective of neural processes underlying behavioral measures of IPD sensitivity. Results showed that the IPM-FR SNR in the 81.6-Hz AM condition was associated with dichotic FM detection thresholds as well as the FM difference score that estimated benefit received from the addition of binaural information in the dichotic FM compared to the diotic FM task. The IPM-FR was able to account for 37% and 40% of the variability in these binaural FM detection measures, respectively. Unlike the IPM-FR, these FM detection tasks were not sensitive to the effects of age in the current study. However, the relationship between the IPM-FR and these binaural FM measures suggests that age-related variability the neural encoding of IPDs may be reflected by performance on these behavioral tasks. Previous studies that established links between the IPM-FR and behavioral IPD sensitivity used stimuli analogous to those used to the elicit the IPM-FR (Haywood et al., 2015; Undurraga et al., 2016; Vercammen et al., 2018). This work expanded on this existing literature by providing evidence that the IPM-FR is also reflective of individual variability in the processing of IPDs produced by dichotic FM.

Speech understanding abilities were assessed using measures of spatial release from masking. The current results showed that IPM-FR magnitude in response to the higher AM stimulus was significantly associated with target-to-masker ratio thresholds in the spatially separated speech-on-speech masking task. Linear regression model predictions of individual performance in this condition were further improved by including average hearing threshold estimates as an additional predictor in the model, such that the model was able to account for approximately 25% of the variance in performance on this task. However, this was not the case when participant age was added as an additional predictor in the model. Similarly, analyses revealed that the relationship between IPM-FR magnitude and spatial release-from-masking thresholds were also likely mediated by the effects of age. In other words, while the variability in the neural encoding of IPD cues was associated with performance on these behavioral speech perception measures, it was not able to account for a substantial amount of variability in performance over what was already accounted for by participant age.

These results are inconsistent with those from Papesh et al. (2017), who assessed relationships between the same spatial release-from-masking measures and the neural sensitivity to changes in IPDs measured using the acoustic change complex. In that study, neural responses were better predictors of spatial release-from-masking thresholds and target-to-masker ratio thresholds in the spatially separated listening condition than participant age or hearing sensitivity (Papesh et al., 2017). Although the IPM-FR and this acoustic change complex both reflect the neural encoding of IPD cues embedded within the temporal fine structure of an AM stimulus, it is possible that differences in the nature of each electrophysiological response may have contributed to these conflicting results. The periodic ±90° IPM used in the current study created a percept of the signal moving from one side to the other. As discussed by Haywood et al. (2015) and Undurraga et al. (2016), these periodic shifts between IPDs leading in the right and left ears in the ongoing stimulus result in modulation of activity in the right and left hemispheres. In contrast, the stimulus used by Papesh et al. (2017) consisted of a single phase shift from a zero to a completely anti-phasic 180° IPD. This stimulus would not periodically modulate the activity of right and left brain hemispheres and would result in a more diffuse intracranial stimulus percept. In addition, Undurraga et al. (2016) argued that a stimulus with a 180° IPD shift like that used by Papesh et al. (2017) may activate neurons in the lateral superior olivary complex of the brainstem that are responsible for processing interaural level differences. These neurons are less likely to be activated by the IPM-FR stimulus used in the current study, which is primarily thought to reflect activity of medial superior olivary complex neurons that are sensitive to IPDs (Undurraga et al., 2016). Therefore, it is also possible that differences in neural activation patterns may have contributed to discrepancies in results between the current study and those of Papesh et al. (2017). Finally, differences in the distribution of participant age as well as the higher 750-Hz carrier frequency used by Papesh et al. (2017) cannot be ruled out as additional factors that may have had an effect on results between these studies. Future research should focus on examining how neurophysiological links between the IPM-FR and speech understanding in spatialized noise are impacted by these factors.

### Effects of Stimulus Parameters on Electrophysiological Responses

#### Effects of AM Rate on the IPM-FR

The current results suggest that changes in stimulus AM rates can impact the IPM-FR. These findings have implications for how stimulus parameters may be optimized to improve IPM-FR measurement reliability as well as to increase sensitivity to participant factors that may contribute to hearing difficulties. The lower AM rate used in the current study is similar to that used in initial studies on the IPM-FR (Haywood et al., 2015; McAlpine et al., 2016; Undurraga et al., 2016), and the higher AM rate is similar to that used in a recent study by Vercammen et al. (2018). However, the current work is the first to directly compare the effects of AM rate on IPM-FRs across the same individuals. Results showed that IPM-FR magnitude was larger in response to the stimulus that was amplitude modulated at 81.6 Hz compared to the stimulus that was amplitude modulated at 40.8 Hz. In addition, while SNRs were not significantly different across AM rate conditions, SNRs calculated from IPM-FRs elicited using the higher 81.6-Hz AM rate tended to be better predictors of behavior than those elicited using the lower 40.8-Hz AM rate. IPM-FRs elicited with the higher AM rate also tended to be more sensitive to the effects of age than those elicited with the lower AM rate. There are several potential explanations for our observed pattern of results. First, the use of a higher AM rate may improve the magnitude of the IPM-FR because it simply contains more AM cycles in the ongoing stimulus than a lower AM rate. This may create more neural responses, or looks, at the ongoing stimulus, which would be expected to result in an increase in neural response strength. A second explanation may be that a steeper modulation slope resulting from the faster 81.6-Hz AM increases neural synchrony, and therefore increases response strength compared to shallower slopes that would occur at lower AM rates. An additional explanation may be that the stronger IPM-FR in the higher AM rate condition results from additional neural responses to energy contained in sidebands that result from the AM of the 500-Hz signal. If these sidebands occur in separate auditory filters and contain IPMs, then participants may essentially be benefiting by an increased number of available stimuli that each contain IPD cues. Finally, while it is known that changes in AM rate impact the activation of ASSR neural generator sites (Giraud et al., 2000; Herdman et al., 2002; Korczak et al., 2012), it is difficult to determine how changes in the activation of these different neural generators with AM rate may also impact the IPM-FR. Future work will attempt to further examine and test these potential explanations to better understand the effects of AM rate on the IPM-FR.

#### Effects of Recording Condition on the ASSR

Auditory steady-state responses were compared to assess recording quality between the dichotic test condition and the diotic control condition that alternated within each recording block. This data quality check is important for the current study because neural responses to the diotic control stimulus were used to calculate IPM-FR SNRs, and any systematic contamination of responses to a particular stimulus would compromise SNR estimations. Results from the current work showed that the 40.8-Hz ASSR was larger in the dichotic test condition compared to the diotic control condition. This was an unexpected finding, given that the only difference between the two stimuli was the addition of periodic IPMs in the temporal fine structure of the ongoing AM stimulus in the dichotic test condition. One possibility is that the higher 40.8-Hz ASSR magnitude observed in the dichotic condition is reflecting the presence of harmonics in the neural response to the IPMs in the stimulus, and do not reflect actual changes in the ASSR. As can be seen in Figure 5A, neural response peaks can be observed at multiples of the 6.8-Hz IPM rate. While these harmonics are reduced in amplitude as frequency increases, it is possible that the 6th harmonic, which would be equivalent to 40.8 Hz, is contributing to the magnitude of the ASSR measured at this frequency. Therefore, the specific stimulus parameters used in the current study may preclude this type of data quality check for the lower amplitude modulated stimulus. This issue is less likely to occur in the higher AM rate condition, as response harmonics that high in frequency are expected to be negligible, as can be observed in Figure 5B. Indeed, 81.6-Hz ASSR magnitudes were not significantly different across the diotic and dichotic stimuli, which suggests that recording quality was comparable across these two conditions.

## Conclusion

The current work confirmed that the IPM-FR is sensitive to the effects of age on the neural encoding of IPD cues. In addition, this study verified that the IPM-FR is reflective of neural processes underlying behavioral IPD discrimination using tests of binaural FM sensitivity. Therefore, these results confirm that the IPM-FR represents a robust tool for the objective assessment of IPD sensitivity. However, further work is required to better understand links between the neural encoding of IPD cues as measured by the IPM-FR and behavioral measures of binaural temporal processing, especially those that assess speech understanding abilities. In addition, future research should continue to investigate the effects of different stimulus parameters on neural and behavioral measures of IPD sensitivity to better understand the effects of age on these responses. The continued development of measures that are sensitive to participant factors that are thought to impact binaural temporal processing and that are also reflective of functional auditory abilities will be integral to the clinical identification and management of auditory difficulties, especially in patients with normal hearing sensitivity.

## Data Availability Statement

The data supporting the conclusions of this article may be made available by the authors upon request.

## Ethics Statement

The studies involving human participants were reviewed and approved by the joint institutional review board of the Department of Veterans Affairs Portland Health Care System and Oregon Health & Science University. The participants provided their written informed consent to participate in this study.

## Author Contributions

CB and FG conceived the study. CB, FG, TK, and RM designed the experiments. TK and RM collected and analyzed the data. CB, FG, TK, and RM interpreted the results. TK drafted the manuscript and revisions. CB, FG, and RM provided critical reviews of the manuscript and approved the final version. All authors contributed to the article and approved the submitted version.

## Conflict of Interest

The authors declare that the research was conducted in the absence of any commercial or financial relationships that could be construed as a potential conflict of interest.

## Footnotes

1 While neural responses from all 30 participants were used to calculate the grand mean spectra illustrated in Figure 5, to facilitate ease of visualization of the IPM-FR and ASSR in each panel, individual responses from one participant were removed from Figures 5B,D due to the presence of 60 Hz electrical noise that was introduced in one recording block when sound booth power was not turned off prior to recording.
